# Deep Microbial Colonization in Saponite-Bearing Fractures in Aged Basaltic Crust: Implications for Subsurface Life on Mars

**DOI:** 10.3389/fmicb.2019.02793

**Published:** 2019-12-05

**Authors:** Yuri Sueoka, Seiya Yamashita, Mariko Kouduka, Yohey Suzuki

**Affiliations:** Department of Earth and Planetary Science, The University of Tokyo, Tokyo, Japan

**Keywords:** water–rock interactions, rock-hosted life, Fe, Mg-smectite, basaltic basement, clay-catalyzed organic synthesis

## Abstract

One of the most promising planetary bodies that might harbor extraterrestrial life is Mars, given the presence of liquid water in the deep subsurface. The upper crust of Mars is mainly composed of >3.7-billion-year-old basaltic lava where heat-driven fluid circulation is negligible. The analogous crustal environment to the Martian subsurface is found in the Earth's oceanic crust composed of basaltic lava. The basaltic crust tends to cool down for 10–20-million-years after formation. However, microbial life in old cold basaltic lava is largely unknown even in the Earth's oceanic crust, because the lack of vigorous circulation prevents sampling of pristine crustal fluid from boreholes. Alternatively, it is important to investigate deep microbial life using pristine drill cores obtained from basaltic lava. We investigated a basaltic rock core sample with mineral-filled fractures drilled during Integral Ocean Drilling Project Expedition 329 that targeted 104-million-year-old oceanic crust. Mineralogical characterizations of fracture-infilling minerals revealed that fractures/veins were filled with Mg-rich smectite called saponite and calcium carbonate. The organic carbon content from the saponite-rich clay fraction in the core sample was 23 times higher than that from the bulk counterpart, which appears to be sufficient to supply energy and carbon sources to saponite-hosted life. Furthermore, a newly developed method to detect microbial cells in a thin-section of the saponite-bearing fracture revealed the dense colonization of SYBR-Green-I stained microbial cells spatially associated with saponite. These results suggest that the presence of saponite in old cold basaltic crust is favorable for microbial life. In addition to carbonaceous chondrite, saponite is a common product of low-temperature reactions between water and mafic minerals on Earth and Mars. It is therefore expected that deep saponite-bearing fractures could host extant life and/or the past life on Mars.

## Introduction

The surfaces of Earth-like planets are extensively covered with basaltic lava as a consequence of planetary differentiation (Hazen, 2012; De Pater and Lissauer, 2015). Mars retained active hydrologic systems at the near-surface settings until ~3-billion-years ago (Ehlmann et al., 2011). Unlike on Earth, the organic and inorganic products of ancient basalt-water interactions are known to be preserved under freeze-drying conditions without being altered by tectonic forces (Wordsworth, 2016). It is therefore expected that the signatures of life and/or biomolecules might be discovered from the Martian surface (Onstott et al., 2019). On Earth, old, tectonically undeformed basaltic lava is ubiquitously distributed in the upper oceanic crust (Heberling et al., 2010). The oceanic crust is 100-million-years old on average (Parsons, 1982), as a result of the tectonic recycling into the mantle (Jarrard, 2003). Understanding of microbial life in the old basaltic lava on Earth is important to constrain the habitability of basaltic lava on Mars.

Previously, basaltic lava underneath sediments called basaltic basement has been microbiologically investigated at 3.5- and 8-million-year-old ridge flank systems (Juan de Fuca: Cowen et al., 2003; North Pond: Orcutt et al., 2013). Recent genome-resolved metagenomics analysis of crustal fluid samples collected from 3.5- to 8-million-year-old basaltic basement revealed the metabolic potential that could support lithotrophic microbial life mediating the oxidation of H_2_, Fe(II) compounds, ammonia and reduced S compounds coupled to the reduction of O_2_, nitrate and sulfate (Tully et al., 2018; Smith et al., 2019). Metabolic activities of lithotrophic microbial life have been indicated by characterizing basaltic rocks drilled prior to crustal fluid sampling (Lever et al., 2013; Zhang et al., 2016a,b).

After 10-million-years after the formation of basaltic basement (10 Ma), the heat-driven circulation of crustal fluid becomes weak (Sclater et al., 1980; Hasterok et al., 2011), which causes the technical difficulty in sampling pristine crustal fluid from drilled boreholes. Hence, microbiological investigations need to be performed by using drilled rocks obtained from >10 Ma basaltic basement. Recently, we have investigated microbial life in old basaltic basement by drilling into 104-Ma oceanic crust, where basaltic lava is overlain by a 75-m thick sediment layer (Yamashita et al., 2019). At the site, low organic contents of the entire sediment column render the penetration of O_2_ in seawater from the ocean floor down to the basaltic basement (D'Hondt et al., 2015). It is also important to note that the fluid circulation in basaltic basement is not detected by heat flow measurements at this site (Expedition 329 Scientists, 2011). By characterizing 110- and 122-m deep basaltic rock cores, it has been revealed that the basaltic basement is permeable and reactive with oxygenated seawater to produce a clay mineral structurally and compositionally similar to Fe-rich smectite called nontronite [(CaO_0.5_,Na)_0.3_${\text{Fe}}_{2}^{3+}$(Si,Al)_4_O_10_(OH)_2_·nH_2_O]. As the formation of nontronite is associated with the oxidation of Fe(II) from basalt dissolution coupled to the reduction of O_2_ from seawater, we infer that 104 Ma oceanic crust could sustain lithotrophic life under aerobic conditions (Yamashita et al., 2019).

Nontronite is widely observed on Mars as a result of oxic basalt-water interactions (Chevrier et al., 2007). Under oxygen-deprived conditions, it is considered that fractures/veins are filled with secondary minerals commonly found under anoxic conditions such as pyrite [FeS_2_], Mg-rich smectite called saponite [Ca_0.17_Mg_3_(Si, Al)_4_O_10_(OH)_2_·n(H_2_O)]_5_ and calcium carbonate such as calcite [CaCO_3_] (Teagle et al., 1996). Calcium carbonate is of particular importance, given that calcium carbonate is the prominent product of basalt-seawater interactions to seal fractures/veins in the oceanic crust on Earth (Müller and Dutkiewicz, 2018). Carbonate-bearing basaltic rocks are widely found on Mars (Murchie et al., 2009). In the 104 Ma oceanic crust where nontronite formation has been reported from the 110- and 122-m deep core samples (Yamashita et al., 2019), a 102-m deep basaltic rock core was associated with fractures/veins filled with calcium carbonate. In this study, we investigated mineralogical and microbiological characteristics of a fracture filled with calcium carbonate.

## Materials and Methods

### Sample Collection

The basaltic rock core sample associated with fractures with calcium carbonate was collected at Site U1365 in the South Pacific Gyre during Integrated Ocean Drilling Program (IODP) Expedition 329 (October 9 through December 13, 2010). The 104 Ma basaltic basement is overlain by 75-m thick sediments found to contain an extremely low abundance of microbial cells (D'Hondt et al., 2015). As primary production near the seawater surface was also extremely low, the supply of detrital organic matter for the consumption of O_2_ is very limited in sediments (D'Hondt et al., 2015).

For drilling into the basaltic basement, a rotary core barrel (RCB) coring system was used on the drilling research vessel JOIDES Resolution. After the core recovery, approximately 18 h passed until subsequent microbiological sampling of the rock sample in the cold room. The portion of the rock core sample was fixed overnight with 2% glutaraldehyde in a solution containing 100 mM Tris–HCl (pH 8) and stored in the Tris–HCl solution without glutaraldehyde at 4°C. For mineralogical characterizations, the portion of the rock core sample was ground and powdered on board as described below and stored at −80°C.

### Contamination Check

Fluorescence microspheres (0.5-μm in diameter) were used to monitor contamination. Fluorescence microspheres in a bag placed on the core-catcher were released into drilling fluid upon the start of drilling. The presence of microspheres was inspected before and after cleaning and subsampling steps. First, the untreated exterior removed from the rock core sample using a flame-sterilized hammer and chisel was examined for contamination. Second, 3% NaCl solution was used to wash the rock core surface twice. Third, the rock core surface was slightly heated with a propane torch, and then the interior and exterior of the flamed rock pieces were separated by a flame-sterilized hammer and chisel. All rock pieces subsampled for contamination check were soaked in 25-mL 3% NaCl solution, from which 3-mL of the solutions with microspheres were filtered using 25 mm black polycarbonate filters (0.22-μm pore size) and examined under epifluorescence using an Olympus BX51 microscope (Olympus, Tokyo, Japan).

### Preparation of a Thin Section and Light Microscopy

We prepared a thin section to clarify the mineral composition within rock fractures. A fracture-bearing rock piece was dehydrated twice in 100% ethanol for 5 min, followed by the infiltration of the rock piece with LR White resin (London Resin Co. Ltd., Aldermaston, England) for 30 min. The infiltrated rock piece was solidified in an oven at 50°C for 48 h. After trimming into a 100-μm thin section, the surface was polished with corundum powder and diamond paste. Mineralogical assemblages were observed using an optical microscope (BX51; Olympus) with a charge-coupled device (CCD) camera (DP71; Olympus).

### Scanning Electron Microscopy (SEM)

Using a Hitachi field emission scanning electron microscopy (FE-SEM) S-4500 instrument (Tokyo, Japan), back-scattering electron images were obtained from the thin section coated with carbon. FE-SEM was operated at an emission current of 15 μA and an accelerating voltage of 15 kV. For chemical compositions of mineral phases, energy-dispersive X-ray spectroscopy (EDS) was used according to contrasts of the image corresponding to atomic density.

### Analysis of X-Ray Diffraction (XRD) Pattern

The powder of the rock core was prepared with a flame-sterilized mortar and pestle. The clay-sized fraction in the powder sample was dispersed in distilled and deionized water, centrifuged at 3,000 rpm for 5 min, and then freeze-dried for storage. X-ray diffraction (XRD) pattern analysis was performed to identify phyllosilicate minerals using a RIGAKU RINT-ULTIMA-2100 (Tokyo, Japan) at an operation voltage of 40 kV and an operation current of 30 mA with monochromatized Cu-Kα radiation. To orient the sheet structure, the freeze-dried sample suspended in distilled and deionized water was mounted on a glass slide. The mounted samples were air-dried and treated with ethylene glycolate and subjected to X-ray scanning in a 2θ range of 2–10°. To determine the 060 reflection for clarifying the sheet structure, the randomly oriented sample was examined.

### Organic Carbon Characterizations of the Clay Fraction

The powdered core sample was suspended in sterilized deionized water. The supernatant after centrifugation at 3,000 rpm for 5 min contained the clay fraction. The clay fraction was collected by centrifugation at 10,000 rpm for 10 min. We measured the organic carbon contents of the powdered core sample and the clay fraction using a mass spectrometer (Thermo Electron DELTAplus Advantage; Thermo Fisher Scientific Inc., Waltham, MA) connected to an elemental analyzer (EA1112, Thermo Electron DELTAplus Advantage) through a Conflo III interface. Before the measurement, the sample was heated at 100°C in 3% HCl to eliminate carbonate minerals, washed twice with distilled, deionized water, and dried.

### Microbiological Characterizations of the Thin Section and the Clay Fraction

We developed a new method to clarify the mineral composition and microbial distribution within rock fractures by modifying a protocol established for the localization of endosymbiotic cells in chemosynthetic animals (Nussbaumer et al., 2006). Previously, hybridizations are performed for thin sections of biological tissues with fluorescently labeled oligonucleotide probes and 4′,6-diamidino-2-phenylindole (DAPI). In this study, microbial cells in the thin section of the rock piece were stained with SYBR Green I (TaKaRa). Prior to SEM-EDS analysis, the thin section was incubated in TE buffer containing SYBR Green I for 5 min. After rinsing with deionized water, the thin section was mounted with the antifade reagent VECTASHIELD (Vector Laboratories, Burlingame, CA, USA). SYBR-Green-I-stained microbial cells in the thin section were observed using an epifluorescence microscope (BX51; Olympus) equipped with a charge-coupled device (CCD) camera (DP71; Olympus). We used two ranges of fluorescence between 540 and 570 nm and 570 and 600 nm for the discrimination of microbial cells from mineral-specific fluorescence signals. For a positive control, *Shewanella oneidensis* (ATCC 700500) cultured aerobically in LB Broth (ATCC Mediun 1065) at 30°C was embedded in LR White resin as described above. A 100-μm thick thin section was prepared from the resin block embedded with *Shewanella* cells. For a negative control, a resin block without *Shewanella* cells was used to prepare a thin section. The thin sections for the positive and negative controls were stained with SYBR Green I and examined using the epifluorescence microscope. To visualize individual microbial cells associated with saponite, the portion of the clay fraction was embedded in LR White resin. Three micrometer thick thin sections were prepared using an ultramicrotome (Reichert Ultracut S; Leica, Wetzlar, Germany). The 3-μm thick thin sections were stained with SYBR Green I and examined using the epifluorescence microscope.

## Results

### Rock Core Descriptions

**Figure 1:**
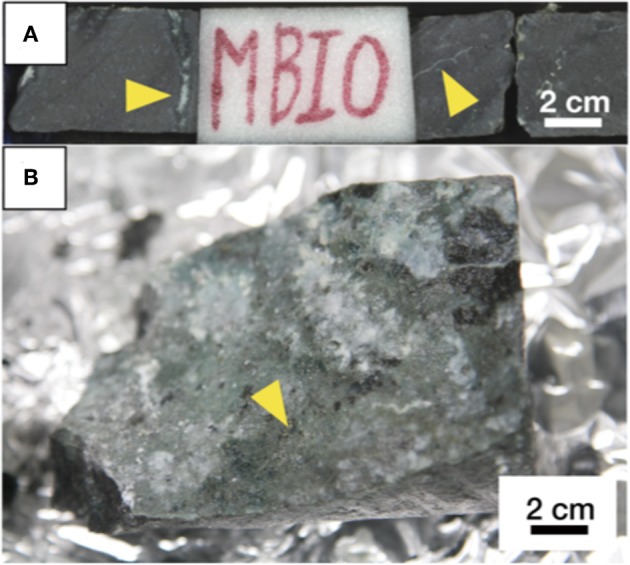
Pictures of a basaltic core sample (U1365E-7R2). **(A)** A whole-round core including the sampling interval as labeled with MBIO. White fractures/veins pointed out by yellow arrows are filled with calcite. **(B)** Fracture surface after opening. A yellow arrow indicates the presence of pyrite grains.

We investigated the basaltic core sample (sample code: U1365E-7R2) at a depth of 102 m below the seafloor (mbsf). Microsphere enumeration revealed that microspheres indicative of microbial contamination from drilling fluid were not detected from the interior of the rock core sample (Supplementary Table 1). On-board visual characterizations of the rock core revealed calcite (white) and celadonite (dark green) were main fracture-infilling minerals (Figure 1A). In addition, small pyrite grains (gold) were visually observed on the opened fracture surface (Figure 1B).

### Characterizations of Fracture-Infilling Minerals

**Figure 2:**
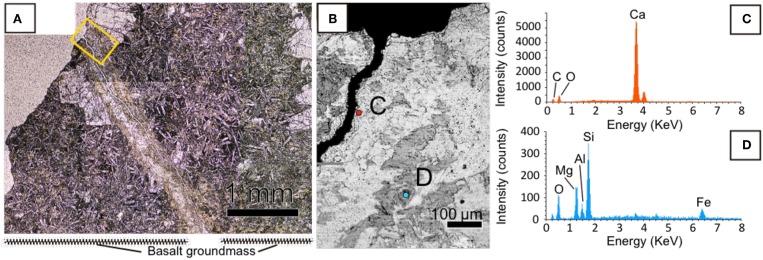
Mineralogical characteristics of a fracture filled with calcium carbonate in U1365E-7R2. **(A)** Optical microscopic image of the fracture with a yellow rectangle indicating an enlarged area shown in **(B)**. **(B)** Back-scattered electron image of the fracture obtained by SEM. **(C,D)** EDS spectra with colors obtained from circles with the same colors in **(B)**.

We prepared and observed a thin section using optical microscopy to investigate the distribution of minerals in a fracture in U1365E-7R2. It was observed that the fracture hosted in phyrytic microcrystalline basaltic groundmass was associated with a white inner portion surrounded by a yellow-brownish outer portion (Figure 2A). We analyzed chemical compositions of the inner and outer portions by SEM-EDS (Figure 2B). As consistent with the on-board visual mineral identification, the white material is calcium carbonate (Figure 2C). From the outer yellow-brownish portion, Si, O, and Mg were detected as major elements, whereas Fe and Al were detected as relatively minor elements (Figure 2D). As a ratio of peak intensities of Si and Mg was ~2:1, it is indicated that the yellow-brownish material is a 2:1 clay mineral.

### Clay Fraction Characterizations of the Rock Core Sample

**Figure 3:**
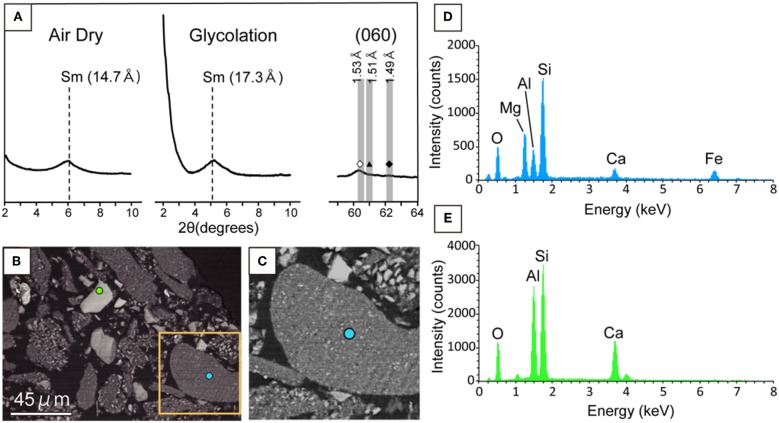
Mineralogical characterization of the clay fraction separated from a powdered core sample in U1365E-7R2. **(A)** Low-angle XRD patterns (2**θ**: 2–10°) from air-dried and ethylene-glycolated samples vertically oriented to the c-axis of phyllosilicate minerals (left and middle). High angle XRD pattern (2θ: 59–64°) including 060 reflections from the randomly oriented sample (right). Sm indicates smectite. In the high-angle XRD pattern, vertical bands show 2θ ranges of 060 reflections from trioctahedral phyllosilicate minerals (left with open diamond), nontronite and celadonite (middle with filled triangle), and dioctahedral phyllosilicate minerals (right with filled diamond; Moslehuddin and Egashira, 1997). **(B)** Back-scattered electron image of the clay fraction with light blue and light green circles, from which EDS spectra were obtained for the low-contrast phase and the high contrast phase **(D,E)**. An orange rectangle indicates an enlarged area shown in **(C)**. **(C)** Back-scattered electron image of the enlarged area in **(B)**. **(D)** EDS spectra from light blue and light green circles in **(B)**.

To identify the yellow-brownish material, a clay-sized fraction was separated from the powdered core sample. XRD analysis of crystallographically oriented clay minerals with and without intercalation using ethylene glycol revealed that based on the expansion of a basal spacing from 1.47 to 1.73 nm after the ethylene glycol treatment (Figure 3A), the clay fraction mainly contained a smectite mineral rather than other non-expandable clays such as mica and chlorite minerals (Moore and Reynolds, 1989). Furthermore, we unambiguously identified the sheet structure of the smectite mineral to be tri-octahedral, based on the 060 reflection (Figure 3A). To clarify the chemical composition of the tri-octahedral smectite, we performed SEM-EDS analysis of the clay fraction. A low-contrast phase characteristic of an EDS spectrum (Figure 3B) similar to that of the yellow-brownish material in the thin section (Figure 2C) was found to include fine grains with a relative high contrast (Figure 3C). EDS spectra obtained from relatively large grains with the same contrast (Figure 3D) as that of the fine grains in the low-contrast phase indicate Ca, Si, Al, and O to be major elements (Figure 3E). Given the presence of anorthite [CaAl_2_Si_2_O_8_] revealed by XRD analysis of the clay fraction (Supplementary Figure 1), it is inferred that the high-contrast phase associated with the low-contrast phase was anorthite. This inference is supported by the fact that Al and Ca peak intensities relative to that of Si were higher in the low-contrast phase in the clay fraction (Figure 3B) than that of the yellow-brownish material in the thin section (Figure 2C). Taken together, the yellow-brownish material found in the fracture was identified to be sapointe, a tri-octahedral smectite mineral with Mg in the octahedral site (Meunier, 2005).

### Organic Carbon Contents of the Bulk and Clay Fraction of the Core Sample

Recently, the abiotic synthesis of amino acids has been reported in saponite formed after serpentinization of olivine and pyroxene minerals at a depth of 173 mbsf in the Atlantis Massif (Ménez et al., 2018). As it is expected that saponite is also enriched in abiotic organic matter in the basaltic basement, we measured the organic carbon content of the clay fraction where saponite was dominantly identified. In comparison to the bulk counterpart before the separation of the clay fraction (an organic carbon content of 0.016 wt. %), the organic carbon content of the clay fraction was 0.363 wt. %. The 23-fold increase in organic carbon content from the saponite-dominated clay fraction is consistent with the localized concentration of amino acids in saponite after serpentinization (a bulk carbon content of 0.023 wt. %; Ménez et al., 2018).

### Distributions of DNA-Stained Microbial Cells in the Mineral-Filled Fracture

**Figure 4:**
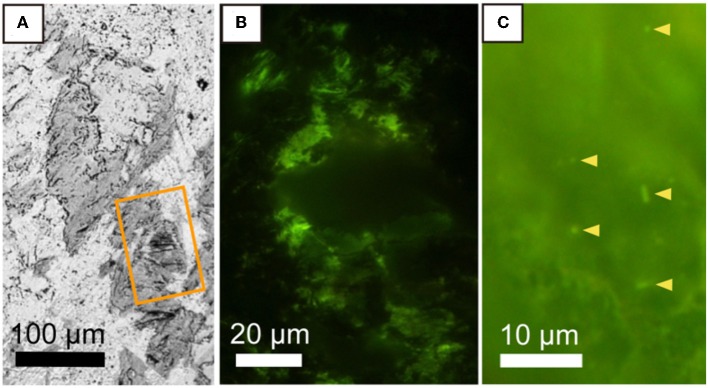
Microbial distribution in a saponite-bearing locus near basaltic groundmass revealed by staining of a thin section with SYBR-Green I. **(A)** Back-scattered electron image of saponite aggregates enlarged from Figure 2B. An orange rectangle indicates the area shown in **(B)**. Fluorescence microscopy images of SYBR Green I-stained microbial cells associated with saponite in a 100-μm thin section of a rock piece **(B)** and in a 3-μm thin section of a clay fraction **(C)**.

We developed a new method to visualize microbial cells in the thin section of the basaltic rock sample by using hydrophilic resin. *S. oneidensis* cells embedded and stained with SYBR-Green I were clearly visualized from the background of resin (Supplementary Figure 2). Thereby, it is possible to correlate the distributions of microbial cells and minerals in fractures. As shown in Figure 4, fluorescence microscopic examination of the saponite-bearing fracture revealed densely colonized microbial cells, where SEM-EDS analysis revealed the presence of saponite near the basalt groundmass (Figure 2C). From these results, we infer that the organic matter enriched in the saponite-bearing fraction is derived, at least in part, from the cellular components of microorganisms. In contrast, DNA-stained microbial cells were not observed neither from basaltic groundmass nor calcium carbonate in the fracture. To demonstrate that microbial cells could be visually distinguishable from saponite, 3-μm thick thin sections of the clay fraction mainly composed of saponite were stained with SYBR-Green I and observed using the fluorescent microscope. As shown in Figure 4C, individual microbial cells with spherical and rod shapes were visualized from the background of saponite particles.

## Discussion

### Temporal and Spatial Variations in Mineral Formation in Basalt Fissures

On Earth, basaltic lava is erupted and solidified at mid-ocean ridges, where oxygenated seawater is vigorously circulated by the buoyance of heated fluid. It is generally known that hydrothermal alterations lead to the formation of secondary minerals such as a mica mineral called celadonite [K(Mg, Fe^2+^)(Fe^3+^, Al)(Si_4_O_10_)(OH)_2_] and iron oxyhydroxides (Teagle et al., 1996; Bach and Edwards, 2003). On the ridge flank associated with the restricted circulation of oxygenated seawater (Lin et al., 2014), pyrite and saponite tend to form in fractures/veins (Teagle et al., 1996; Lever et al., 2013). After the cooling of the ridge flank, further infilling with calcium carbonate is generally considered to seal fractures/veins (Müller and Dutkiewicz, 2018). Recently, we revealed that subseafloor basaltic lava is percolated with oxygenated seawater through fractures/veins filled with celadonite and iron oxyhydroxides in cold oceanic crust aged 33 and 104 Ma (Yamashita et al., 2019). The product of basalt-water interactions under oxic conditions is nontronite associated with Fe(III) in the octahedral layer.

In U1365E-7R2, the formation of nontronite was not evident at the interface between saponite and basalt groundmass where nontronite has been found in the deeper basaltic core samples with fractures/veins filled with celadonite and iron oxyhydroxides (Yamashita et al., 2019). This result suggests that the oxygenated seawater is not intruded in the fracture through the interface adjacent to saponite. The persistence of pyrite in the calcite-filled fracture also supports the possibility that the oxygenated seawater is not permeable in the calcite-filled fracture. The mineral assemblage and texture observed in the fracture support the following mineralization order:

Stage 1: Celadonite formation from high-temperature crustal fluid around the mid-oceanic ridge (< ~1 Ma).

Stage 2: The formation of saponite and pyrite from moderate-temperature crustal fluid under reducing conditions at the ridge flak (< ~10 Ma).

Stage 3: The formation of calcite from low-temperature crustal fluid from the ridge flank to the abyssal plain (< ~10–20 Ma).

### Saponite-Hosted Organic Matter and Microbial Cells

DNA-stained microbial cells were densely observed where saponite was distributed in the fracture. This spatially limited occurrence excludes the possibility that contaminant microbial cells were attached during polishing the thin section. In addition to microspheres checked for microbial contamination (Supplementary Table 1), bentonite used in the drilling fluid was different from saponite (Yamashita et al., 2019). It is therefore unlikely that microbial cells were introduced into the fracture during drilling. The enrichment of organic matter in the saponite-bearing fraction is consistent with the dense colonization of DNA-stained cells with saponite. As saponite has the ability to effectively adsorb organic matter (Pinnavaia, 1983; Ménez et al., 2018), it is plausible to observe DNA-stained cells in the fracture-infilling saponite. The sources of organic matter and microbial cells might be explained by four cases:

Case 1: Organic matter and microbial cells on saponite are derived from seawater. In this case, both seem to originate from the photosynthetic biosphere.

Case 2: Organic matter on saponite is derived from seawater, and microbial cells are growing *in situ* by metabolizing the organic matter. In this case, microbes are dependent on photosynthetic organic matter.

Case 3: Organic matter and microbial cells are produced on saponite *in situ*. In this case, energy sources for lithoautotrophic metabolisms from saponite are unknown. One possibility could be H_2_ derived from H_2_O reacted with Fe(II) in saponite. This reaction has been demonstrated by Fe(II) in octahedral layers in Fe(II)-rich chlorites at high temperatures (Lempart et al., 2018).

Case 4: Organic matter is produced abiotically, and microbial cells metabolize the organic matter. In this case, abiotic organic synthesis appears to be catalyzed by Fe(II)-rich saponite with H_2_ as the energy source (Ménez et al., 2018). H_2_ might be derived from the moderate-temperature crustal fluid at the Stage 2.

There is the possibility that DNA-stained cells are the relics of microbial life metabolically active after the saponite formation and before the infilling of calcium carbonate (~1 to ~20 Ma). This possibility seems to be supported by the tight sealing of the fracture with calcium carbonate and saponite, which might have prevented the hydrolysis of DNA in fossilized cells. However, the life time of DNA is the order of, at most, several million years in geological formations (Allentoft et al., 2012), and the persistence of DNA for ~80 Ma is unlikely. Hence, it is plausible that microbial cells could survive under oxygen-deprived conditions by metabolizing inorganic and/or organic energy available around saponite.

### Implications for Extant and Past Life in the Subsurface on Mars

The abundant and widespread occurrence of saponite has been found on the crater walls in ~4-billion-year-old basaltic terrains. These findings lead to the inference that until ~3-billion-years ago, there appears to have been near-neutral basalt-water interactions in the deep subsurface on Mars (Murchie et al., 2009; Ehlmann and Edwards, 2014). As the supply of atmospheric oxidant was periodically limited (Hurowitz et al., 2017), the deprival of O_2_ by reacting with Fe(II) favors the production of saponite over nontronite in the ancient basaltic subsurface (Bach, 2016). The production of saponite is also expected in the modern basaltic crust where the persistence of rock-water interactions has been recently demonstrated (Wade et al., 2017; Orosei et al., 2018). Given the prevalence of organic matter associated with saponite in the Solar System (Pearson et al., 2002), it is indicated that saponite-bearing fractures/veins in the basaltic crust could host extant microbial life and/or signatures from past life on Mars.

## Conclusion

We revealed that microbial cells were hosted in saponite-bearing fractures. Given the saponite-bearing fraction enriched with organic matter, it is likely that the dense colonization of microbial cells is supported by lithotrophy and/or heterotrophy. Given that low-temperature interactions between mafic minerals and water universally result in the assemblage of saponite and organic matter, extraterrestrial life could be found in the subsurface, where liquid water is disseminated in rock bodies containing mafic minerals.

## Data Availability Statement

All datasets generated for this study are included in the article/Supplementary Material.

## Author Contributions

YSue conducted SEM-EDS and microbial cell characterizations and wrote the manuscript. SY conducted optical microscopy and XRD. MK conducted microbial cell characterizations. YSuz initiated and planned the project, conducted sampling, and wrote the manuscript.

### Conflict of Interest

The authors declare that the research was conducted in the absence of any commercial or financial relationships that could be construed as a potential conflict of interest.
