# Supplementary material for: Deep Microbial Colonization in Saponite-Bearing Fractures in Aged Basaltic Crust: Implications for Subsurface Life on Mars
Source: Front Microbiol. 2019 Dec 5;10:2793. doi: 10.3389/fmicb.2019.02793 (PMC6906187; doi:10.3389/fmicb.2019.02793)
Supplement: Supplementary file 1 [file Data_Sheet_1.docx]

Supplementary Material

**Supplementary Table 1.** Results of contamination check for U1365E-7R2. The minimum detection limit was ~100 microspheres/cm^3^ rock.

| Microsphere counts (Log_10_ microsphere cm^−3^) | | | |
| --- | --- | --- | --- |
| Untreated core | Exterior washed | Exterior flamed | Interior |
| 3.7 | 2.3 | 2.3 | ND |

ND: no microspheres were observed by microscopic observations.

**
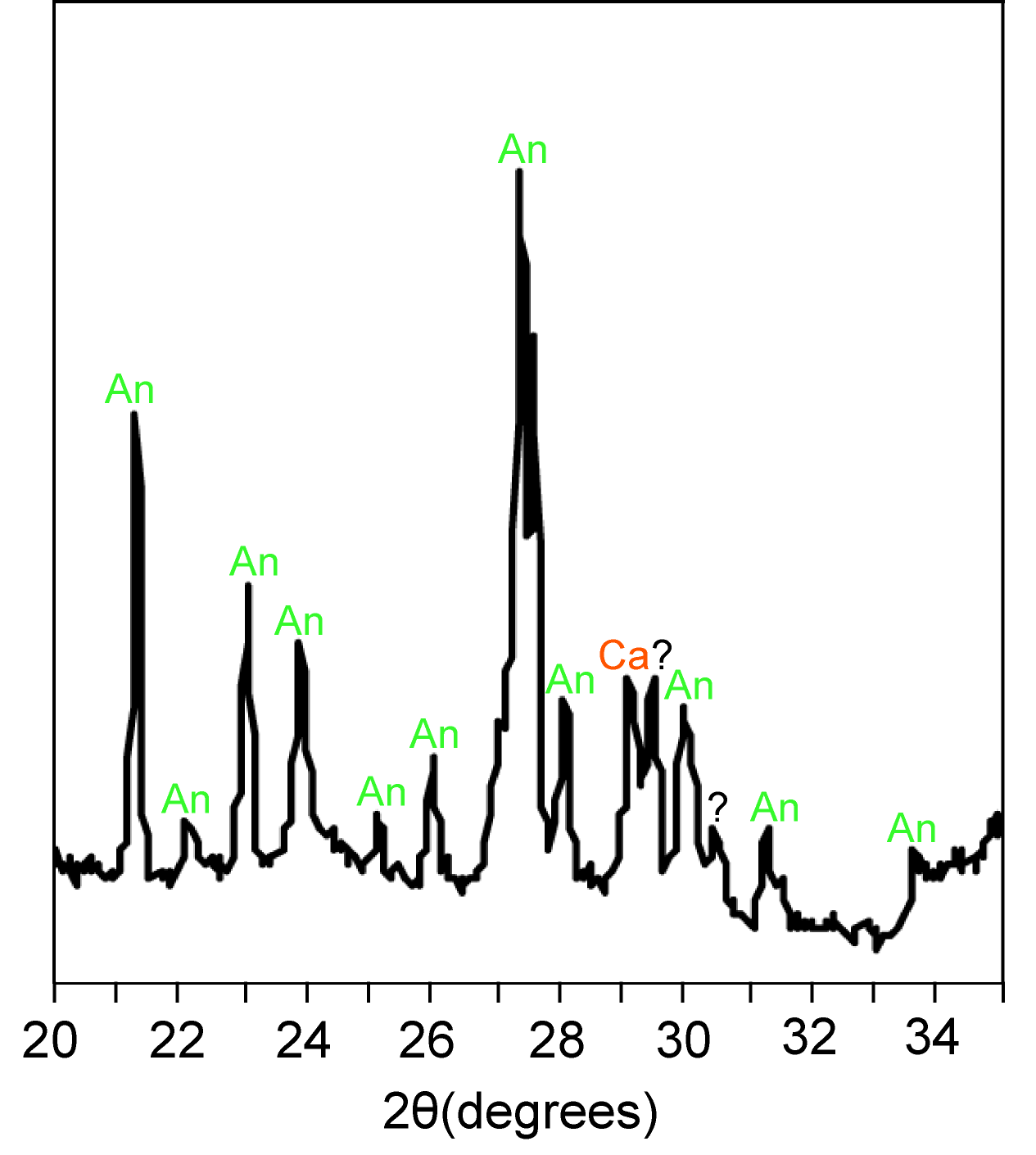
**

**Supplementary Figure 1.** XRD pattern of air-dried clay fraction of U1365E-7R2 (2*θ*: 20-35°). An and Ca indicate anorthite [CaAl_2_Si_2_O_8_] and calcite [CaCO_3_].


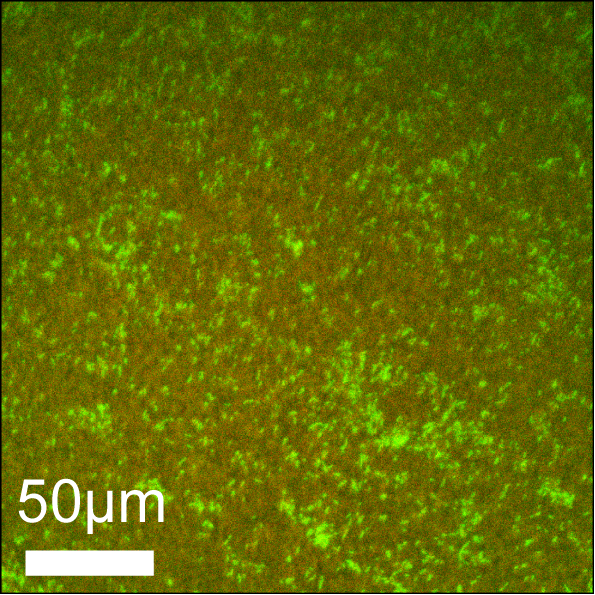


**Supplementary Figure 2.** *Shewanella oneidensis* embedded in LR White resin. SYBR Green I was used to stain cellular DNA.
